# Co-Occurrence of Aflatoxin B_1_, Zearalenone and Ochratoxin A in Feed and Feed Materials in Central Italy from 2018 to 2022

**DOI:** 10.3390/foods13020313

**Published:** 2024-01-18

**Authors:** Stefano Sdogati, Tommaso Pacini, Rita Bibi, Angela Caporali, Emanuela Verdini, Serenella Orsini, Roberta Ortenzi, Ivan Pecorelli

**Affiliations:** Istituto Zooprofilattico Sperimentale dell’Umbria e delle Marche “Togo Rosati”, 06126 Perugia, Italyi.pecorelli@izsum.it (I.P.)

**Keywords:** aflatoxin B_1_, zearalenone, ochratoxin A, occurrence, feed, maize

## Abstract

Mycotoxin contamination of feed and feed materials represent a serious health hazard. This study details the occurrence of aflatoxin B_1_ (AFB_1_), zearalenone (ZEN) and ochratoxin A (OTA) in 826 feed and 617 feed material samples, collected in two Italian Regions (Umbria and Marche) from 2018 to 2022 analyzed using a UPLC-FLD platform. The developed method was validated and accredited (ISO/IEC 17025) with satisfactory accuracy and precision data obtained in repeatability and intralaboratory reproducibility conditions. Feed had a higher incidence of contaminated samples (26%) with respect to feed materials (6%). AFB_1_ was found up to 0.1045 mg/kg in cattle feeds and 0.1234 mg/kg in maize; ZEN was detected up to 6.420 mg/kg in sheep feed while OTA was rarely reported and in lower concentrations (up to 0.085 mg/kg). Co-contamination of at least two mycotoxins was reported in 0.8% of the analyzed samples. The incidence of above maximum content/guidance level samples was 2% for feed and feed materials while almost 3-fold-higher for maize (5.8%) suggesting how mycotoxin contamination can affect some matrices more than others. Obtained data can be useful to improve official monitoring plans and therefore further raise awareness of this issue between agriculture stakeholders, healthcare entities and non-professionals.

## 1. Introduction

Mycotoxins are secondary metabolites produced by a plethora of fungi species, such as *Aspergillus*, *Fusarium*, *Penicillium*, *Stachybotrys* and others; they are able to cause toxic responses at low concentrations when introduced in higher vertebrates’ organisms [1]. Up to 300 mycotoxins are identified and characterized at the present time but only a handful possess some relevance for animal and human health [2]. Relevant mycotoxins can be divided in five families: aflatoxins (AFs), ochratoxins (OTs), fumonisins (FUMs), trichothecens (TCs) and zearalenone (ZEN). Mycotoxins exert their toxicity through a wide range of different mechanisms. *Aspergillus flavus* and *Aspergillus parasiticus* are the most common producers of the aflatoxin family (aflatoxin B_1_, B_2_, G_1_ and G_2_). Aflatoxins are bioactivated by cytochrome metabolism into a reactive intermediate (exo-aflatoxin B_1_-8,9-epoxide) responsible for DNA adduction leading to DNA damage during replication [3]. Aflatoxins mainly target the liver leading to loss of organ function (icterus) reduction of serum protein synthesis, coagulopathy and necrosis. Ochratoxins are produced by *Aspergillus* and *Penicillium* fungi strains and mechanisms of action are yet to be elucidated, although structure similarity with phenylalanine suggests an antagonist effect on the biological targets of this aminoacid leading to calcium homeostasis imbalance and reduced protein synthesis [4]. Among *Fusarium* toxins, zearalenone promotes prolactin and luteinizing hormone (LH) release that interferes with the regular estrus cycle, ovulation and embryo implantation. Animal exposure to mycotoxins usually happens through ingestion of contaminated feed leading to clinical and subclinical mycotoxicoses [5]. Animal response to mycotoxin exposure varies between species with sheep being relatively resistant compared to poultry, piglets and cattle [6]. Dogs are also quite sensitive to aflatoxins with a median lethal dose (LD_50_) of 0.5–1.5 mg/kg body weight (bw) for AFB_1_ [7]. Aflatoxicosis outbreaks, via ingestion of contaminated dog food, were periodically reported in literature with an animal death rate, in some cases, around 90% [8,9,10,11]. Swine are known to be among the most susceptible species to zearalenone exposure especially when looking at young specimens and sows. Cattle and sheep appear to be far more resistant to estrogenic side effects of zearalenone even allowing the use of zeranole, a chemical analogue of this mycotoxin, as an animal growth promoter [12]. Ochratoxin exposure causes nephrotoxicity in laboratory animals with LD_50_ ranging from 8.1 to 30.3 mg/kg bw depending on the species. Swine and poultry are the most sensitive farm animals to ochratoxins: swine may develop a particular condition called porcine nephropathy, which is thought to be endemic of North European countries [13]; poultry, especially ducklings, show alteration in the intestinal microbiota, reduction in meat pigmentation and egg shell fragility as a consequence of reduced food intake [14,15]. Mycotoxin contamination strongly impact the global economy forcing farmers to destroy infested crops, decreasing animal productivity and increasing the costs of animal treatment [16]. Humidity and temperature are the main environmental parameters concurring in the development of fungi invasions: optimal conditions are often met in tropical and subtropical climates [17,18] although, as the effects of climate change (prolonged droughts, extreme rainfalls, rising of temperatures and carbon dioxide levels), are starting to affect continental climates as well, this issue is going to become a worldwide challenge in a matter of time. Mycotoxins can directly influence the health of people: consumption of contaminated foods led, in 1995, to severe outbreaks in India where thousands of people suffered of acute mycotoxicosis in the Kashmir valley. More recently, 20 people died after the ingestion of aflatoxin and fumonisin highly contaminated maize in Tanzania [19,20,21]. Human exposure may also occur via transfer of mycotoxins from the contaminated crop, through the animal metabolism, to the final animal by-product. Zearalenone and Ochratoxin A (OTA) were found in eggs, milk, cheese and organ meats [22,23,24,25,26,27,28,29,30]. The most remarkable example of feed-to-food transfer is the extensively studied occurrence of aflatoxin M_1_ (AFM_1_), a hydroxylated metabolite of aflatoxin B_1_ (AFB_1_), in milk and cheese [31,32]. AFB_1_ is recognized as the most potent naturally occurring carcinogen [33] and aflatoxin M_1_ was recently classified as a group 1 human carcinogen [34]. To protect human and animal health, the European Commission laid down several regulations on food and feed sampling [35,36] together with relevant maximum residue levels (MRLs), reported in the Commission’s Regulation (EC) N° 915/2023 [37] and maximum contents in Directive 2002/32/EC [38], respectively. In addition, a recommendation establishing guidance levels for deoxynivalenol, zearalenone, ochratoxin A, fumonisins B_1_ and B_2_, T-2 and HT-2 toxins in feed materials and compound feed was published in 2016 [39]. Thorough monitoring programs of food, feed and feed materials are pivotal to avoid economic losses linked to mycotoxins animal exposure. Through the years, the scientific community developed a plethora of different analytical techniques for mycotoxins determination: thin layer chromatography (TLC), capillary electrophoretic immunoassay (CEIA), enzyme-linked immunosorbent assay (ELISA), gas-chromatography coupled to electronic capture detection (GC-ECD), flame ionization detector (GC-FID), UV detector (GC-UV), mass spectrometry (GC-MS/MS) and liquid chromatography coupled with mass spectrometry (LC-MS and LC-MS/MS) are the most common [40,41,42,43,44]. Liquid chromatography coupled with fluorescence detection (LC-FLD) is a valid alternative with a strong literature, supporting the analytical determination of these substances in food and feed [45,46,47]. Mycotoxins often possess fluorophore groups in their scaffold allowing a sensitive and specific determination using LC-FLD instrumentations. The present work details the analysis of 1443 samples (826 feed and 617 feed materials) during a five-year period (2018–2022). Samples were collected in the Umbria and Marche Regions (central Italy) and analyzed applying an in-house developed, validated and accredited UPLC-FLD methodology in order to elucidate the occurrence and contamination levels of aflatoxin B_1_ (AFB_1_), zearalenone (ZEN) and ochratoxin A (OTA) in feed and feed ingredients in the Italian market. The main goal of this work is evaluating the level of contamination in feeds, destined to different animal species, and different types of feed materials using a reliable methodology. Obtained data can be useful to institutional healthcare entities to highlight current issues in terms of mycotoxin contamination of feeds and feed materials and providing to institutional healthcare entities useful information to adjourn and develop precise monitoring plans. Relevant maximum contents and guidance levels for AFB_1_, ZEN and OTA are reported in Table 1.

## 2. Materials and Methods

### 2.1. Sample Collection

A total of 1443 samples (826 feed and 617 feed materials) were analyzed from 2018 to 2022. Feed and feed materials were collected within the frameworks of the Umbria and Marche Regions Animal Welfare Control Plan and National Animal Nutrition Plan for mycotoxin surveillance and monitoring. Feed samples were organized by animal species (cattle, cow, dog, swine, sheep and rabbit) while feed materials were organized by type (barley, maize, oat, triticale and wheat). Less represented matrices, sampled only few times throughout the years, like cervid feed, horse feed, cottonseed, rice, sorghum and soy were unified under “other feed species” and “other feed materials” respectively. Collected samples were finely milled with dry ice using a Retsch Grindomix GM300 (Haan, Germany) and stored at −20 °C. Considering the non-homogeneous distribution of most mycotoxins in feed, samples were transformed in slurry on the day of the analysis with the addition of a given amount of water in order to obtain a homogenous suspension. Maize and wheat samples were slurred using a sample/water ratio of 0.8 (e.g., 1000 g of sample + 800 mL of water) while for feed and other raw materials the sample/water ratio was 1.6. Lippolis and coworkers pointed out that slurry preparation is a better choice, compared to dry ice milling, in order to obtain accurate and more precise analytical results [48].

### 2.2. Standards and Reagents

Aflatoxin B_1_, zearalenone and ochratoxin A reference materials solutions were purchased by Lab Instruments S.R.L. (Castellana Grotte, Italy). HPLC grade methanol (MeOH) and acetonitrile (ACN) were obtained from Carlo Erba Reagents S.R.L. (Milan, Italy). Water was purified using a Milli-Q system (Millipore, Merck KGaA, Darmstadt, Germany). Analytical grade acetic acid (AcOH), sodium chloride (NaCl), potassium chloride (KCl), sodium phosphate monobasic (Na_2_HPO_4_), potassium dihydrogen phosphate (K_2_HPO_4_), hydrochloric acid (HCl) and sodium hydroxide (NaOH) were obtained from Sigma-Aldrich (St. Louis, MO, USA). VICAM AOZ LC #G1031 immunoaffinity columns (IACs) were obtained from Waters (Milford, DE, USA). Hydrophilic PTFE 0.22 µm filters were purchased from AISIMO LTD (London, UK).

### 2.3. Sample Preparation

The sample preparation procedure was developed following Gobel and coworkers procedure [49] with few modifications. Slurred samples were weighed in a 250 mL PPCO centrifuge bottle: maize and wheat samples (45 g of slurry, corresponding to 25 g of sample) were extracted with 80 mL of MeOH while feed and other feed materials (65 g of slurry, corresponding to 25 g of sample) were extracted with 60 mL of ACN. Samples were vortexed, stirred on a horizontal shaker for 30′ and then centrifuged at 2042 RCF (Relative Centrifuge Force) for 15′ at 22 °C, 10 mL of the centrifuged extract were diluted with 40 mL of PBS buffer (pH 7.0). pH of the diluted solution was checked and, if necessary adjusted, to 7.0 ± 0.1 with NaOH or HCl 1M solutions. For feed analysis, 10 mL of diluted extract were loaded in the VICAM IACs; for feed materials the loading volume was 5 mL. The diluted sample was passed through the IACs at a speed of 1–2 drops per second; after that, 10 mL PBS buffer (pH 7.0) and 10 mL H_2_O were added to wash the columns. Elution of mycotoxins was performed loading 2 mL of MeOH followed by 1.5 mL of AcOH 0.1% collecting the eluate in a glass tube. Sample were dried using a thermostated nitrogen evaporator set to 45 °C. After evaporation, feed samples were reconstituted with 0.5 mL of ACN/AcOH 4% 50:50 (*v*/*v*) mixture while feed materials were reconstituted with 1 mL of the same mixture. Samples were finally vortexed, filtered through 0.22 µm PTFE filters and injected in the UPLC system.

### 2.4. UPLC-FLD Method

Chromatographic separation was performed using a UHPLC Shimadzu Nexera X2 (Kyoto, Japan) equipped with a fluorescence detector (RF-20AXS). Analytes separation was performed using a Kinetex C18 (50 × 2.1 mm; i.d. 2.6 µm) equipped with a column guard both provided by Phenomenex (Torrance, CA, USA). Column oven temperature was set to 30 °C and injection volume to 5 µL. Mobile phases were MeOH (A), ACN (B) and AcOH 1% (C) and the initial flow was set to 0.6 mL min^−1^. The developed method detailed gradient, as well as FLD parameters, are reported in the Supplementary Materials (Table S1). 

### 2.5. Method Validation

**Figure 1:**
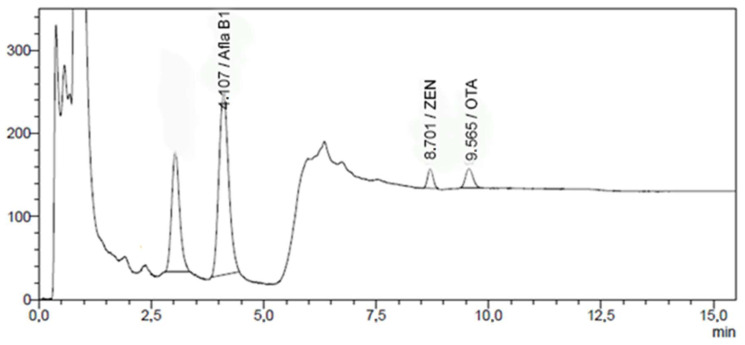
Certified Reference Material for aflatoxins (ERM-BE375) spiked with ZEN and OTA at LOQ level.

An unambiguous regulation establishing performance criteria of analytical methods for mycotoxins determination in feed is not available at the present time, therefore method performance parameters such as specificity, linearity, limit of detection (LOD) and quantitation (LOQ), accuracy and precision and repeatability (RSD_r_) and intralaboratory reproducibility (RSD_wR_) conditions were evaluated considering alternative normative sources. Specificity, in terms of tolerance of analyte retention time (RT), was evaluated in accordance with document SANTE/12089/2016 [50]. Linearity was evaluated following paragraph C17 of document SANTE/11312/2021v2 [51] for AFB_1_ at 0.010, 0.025, 0.050, 0.100 and 0.200 µg/mL; for ZEN at 0.030, 0.125, 0.313, 0.625 and 1.250 µg/mL; and for OTA at 0.013, 0.031, 0.063, 0.125, 0.250 and 0.500 µg/mL considering the non-analytical point 0:0. OTA linearity was also assessed at 0.003, 0.013, 0.031, 0.063, and 0.125 µg/mL for dog feed analysis. Theoretical LOD and LOQ values were determined, based on calibration curves, as reported in paragraph 6.3.2 and 7.3.2 of “Validation of Analytical Procedures: Text and Methodology, Q2(R1)” [52]. Practical LOQs were slightly modified on the basis of current MRLs. Validation was carried out considering feed for different animal species (dairy cows, swine, poultry, horses and dogs) and the most common feed materials used in animal feeding (maize, rapeseed and barley). In summary, precision (RSD_r_) and accuracy (recovery) in repeatability conditions, were evaluated analyzing nine spiked or certified reference materials (CRMs) feed and feed material samples at different levels. The first validation level concentration coincides with the practical LOQ of the method for each relevant mycotoxin. Spiking levels were chosen based on the MRLs or guidance values reported by current legislations [38,39]. A chromatogram of a Certified Reference Material (ERM-BE375) (Aflatoxin B_1_: 0.0026 mg/kg) spiked with ZEN (0.050 mg/kg) and OTA (0.025 mg/kg) is reported in Figure 1.

Intralaboratory reproducibility data (RSD_WR_) was obtained during routine analysis evaluating spiked samples recoveries at LOQ of twenty independent analytical batches. Finally, method accuracy was also checked by participating to three Proficiency Tests from 2019 to 2022. Precision and accuracy performances were evaluated on the basis of performance criteria for confirmatory methods reported in Commission Regulation (EC) N° 401/2006. Relevant validation data are reported in Table 2 and Table 3 while additional information on linearity, LODs, LOQs and Proficiency Test (PTs) results are reported in Supplementary Materials Tables S2–S4. 

## 3. Results

### 3.1. Method Validation

Method specificity was checked comparing analytes’ RT in spiked or incurred samples with the average RT values of standard solutions [50]. Specificity parameters, for each analyte, were met if RTs of spiked/incurred samples was inside a ± 0.1 min RT acceptance window around the average RT of calibration standards. The calibration curves’ linearity was compliant with chapter C17 of the guidance document SANTE/11312/2021v2. Back-calculated concentrations (BCC) and the correspondent deviations from true concentrations were always <20% (Table S2). Theoretical LODs and LOQs are calculated as 3.3 × S_b_/b and 10 × S_b_/b, respectively, where S_b_ is the residual standard deviation of a regression line and b is the slope of the calibration curve. Starting from the theoretical LOQs values and taking into account the MRL_S_ stated for each mycotoxin, practical LOQs were slightly modified and validated as reported in Table 2 and Table 3 [52]. Practical LOQs were established at 2/5 of the lowest MRL for AFB_1_ in feed and feed materials (Table 1) and for OTA (guidance levels) in cereal-based dog food and cereal products; for ZEN and OTA in feed at 1/2 of the lowest guidance level reported in the Commission Recommendation N° 1319/2016. Finally, as the lowest guidance level for ZEN in feed materials was set at 2 mg/kg (cereals and cereal products with the exception of maize by-products), practical LOQ was set at 1/10 of said level [53]. In repeatability conditions, recoveries were between 66% and 103% for AFB_1_, between 76% and 109% for ZEN while for OTA between 60% and 97% with RSD_r_ ranging from 3.0 to 12 for AFB_1_, from 2.6 to 17 for ZEN and from 1.1 to 12 for OTA. In intralaboratory reproducibility conditions recoveries were in the range of 72–92% while RSDw_R_ ranged from 7.5 to 22. These performances met the “Specific requirements for confirmatory methods” reported for AFB_1_, ZEN and OTA in the Commission Regulation (EC) No. 401/2006. Method performances were evaluated through participation to interlaboratory studies organized by different international organizations such as Food Analysis Performance Assessment Scheme (FAPAS) and Progetto Trieste Proficiency Testing Scheme. In all cases, z-scores were considered satisfactory (|z|: ≤ 2) (Table S4). The method was finally accredited following ISO/IEC 17025 [54].

### 3.2. Occurrence in Feed and Feed Materials Samples

A total of 826 feed samples and 617 feed materials were collected from 2018 to 2022. Collected samples were categorized by animal species or type of feed material and analyzed according to chapter 2.3. Sheep (31.6%), cattle (23.5%) and swine feed (21.7%) samples accounted for almost 80% of the collected samples followed by poultry (7.5%), cow (6.5%), other species (3.8%), dog (3.1%) and rabbit feed (2.3%). Among feed materials maize samples were the most collected (38.9%) followed by barley (30.1%), wheat (9.9%), other materials (9.1%), oat (7.1%) and triticale (4.9%). Two hundred eleven feed samples (26% of the total) were contaminated by at least one mycotoxin above LOQ; among these, 16 samples had one of the selected mycotoxins above the maximum content or guidance value (Table 4). The highest incidence of contaminated samples was reported for rabbit feeds (53%), although sample population was small (*n* = 19), followed by swine (35%) “other species” (29%) and cow feed (28%). Interestingly, mycotoxins were never detected in dog feed. Contamination incidence in feed materials was 5.8% (36 out of 617 samples). Thirty-five out of thirty-six contaminated feed materials were maize samples. Maize was basically the only feed material matrix with detectable mycotoxins although incidence was generally lower than feeds (15%). Overall incidence of feed and feed materials from 2018 to 2022 was reported in Figure S1.

ZEN was found in 139 out of 826 samples followed by AFB_1_ (77) and OTA (6). Eleven samples contained more than one mycotoxin. AFB_1_ was responsible for maize contamination in 97% of the cases. Barley, oat, triticale and wheat samples were always free of quantitable mycotoxins.

Seventy-seven feed samples contained AFB_1_ with levels ranging from 0.0021 to 0.1045 mg/kg with an average concentration of 0.0084 mg/kg. The highest AFB_1_ levels were detected in two cattle feeds with 0.0985 and to 0.1045 mg/kg, respectively (Table 5). In addition, other two samples were above the maximum content according to current regulation: a poultry feed (0.0291 mg/kg) and a sheep feed sample (0.0216 mg/kg). Cattle, swine and poultry feed samples had the highest incidence (14%, 13%, and 8% respectively) compared to the other types of feeding. In cattle feed, the average concentration of AFB_1_ was the highest (0.0133 mg/kg), followed by poultry (0.0107 mg/kg), cow (0.0072 mg/kg), sheep (0.0061 mg/kg) swine (0.0045 mg/kg) and other species feed (0.0031 mg/kg).

AFB_1_ levels in feed materials ranged from 0.0085 to 0.1234 mg/kg (average: 0.0349 mg/kg). Average concentration in maize was almost three-fold higher than cattle feed, therefore suggesting that this matrix is much more susceptible to heavy mycotoxin contamination compared to other feed materials and feeds.

More importantly, 14 out of 35 incurred maize samples showed AFB_1_ levels higher than the maximum content permitted by the European regulation (0.02 mg/kg).

Zearalenone levels ranged from 0.051 to 6.420 mg/kg with an average concentration of 0.409 mg/kg in feed. On average, rabbit (0.765 mg/kg) sheep (0.602 mg/kg) and cattle feed (0.527 mg/kg) were the most ZEN contaminated matrices; other types of feed showed concentrations up to three times lower than the average.

Occurrence data shows that ZEN is usually found at low levels although, in some cases, its concentrations may rise up to 16 times higher than the average concentration. ZEN incidence is generally higher than AFB_1_ especially in swine and cow feed (up to 25%). Rabbit feed samples showed the highest incidence (47%) although the sample population was extremely small (*n* = 19). Only in one feed material (maize sample), ZEN was detected above LOQ (0.668 mg/kg) but this concentration was several times lower than the guidance limit set for this type of matrix (3 mg/kg). Non-compliant samples were 14 out of 826 (1.6%) containing ZEN levels that were above the ones permitted by the guidance [39]. Among these, more than half (9) were sheep feed samples suggesting how this type of feed may be more susceptible to ZEN contamination. Extremely high levels of ZEN were also occasionally detected in cattle (up to 6.420 mg/kg), swine (up to 1.698 mg/kg) and rabbit feed (up to 5.723 mg/kg). For these specific species, guidance levels have not yet been set (e.g., rabbit) and, if established, they only refer to young specimens such as piglets and calves [39]. Ochratoxin A was rarely detected in feed samples (0.7%) ranging from 0.034 to 0.042 mg/kg in cattle feed (average: 0.039 mg/kg) and from 0.038 to 0.085 mg/kg in sheep feed (average: 0.061 mg/kg). Guidance levels for OTA in cattle and sheep feed have not yet been set at the present time. Low concentrations and low incidence rates suggest that OTA contamination is, in this precise case, a marginal issue. OTA was never detected in feed materials.

## 4. Discussion

### 4.1. Aflatoxin B1

Several scientific studies were carried out in the recent years to evaluate the occurrence of these mycotoxins in feed and feed materials. Dimitrieska-Stojković and colleagues analyzed 67 samples intended for dairy cows’ consumption (22 maize and 45 feed samples), they reported a non-compliant incidence of 13.4% [55]. Average concentration in maize was 0.0221 mg/kg while in feed (complementary and silage) it was 0.0101 mg/kg. Iqbal and coworkers analyzed 24 poultry feed samples reporting an average AFB_1_ contamination around 0.006 mg/kg [56]. Zhao and coworkers analyzed 1417 feed samples from 2018 to 2020 reporting AFB_1_ average levels varying from 0.0035 to 0.0154 mg/kg. Maximum levels in feed were up to 0.0775 mg/kg. Moreover, among the 2090 feed ingredients analyzed, maize was the most contaminated (up to 0.2210 mg/kg) [57]. Data reported by these authors are consistent with our monitoring study, in terms of incidence and average AFB_1_ concentrations, although other literature reports slightly different findings [58,59]. 

### 4.2. Zearalenone

Mahato and colleagues [60] reviewed several studies investigating ZEN occurrence in food and feed, registering a great variability in terms of concentration levels. For example, Chang and coworkers [61] analyzed 653 feed and feed ingredients in South Korea during an eight-year period with mean concentration values ranging from 0.032 to 0.134 mg/kg; only four samples exceeded EU guidance limits. In contrast, Zhao and coworkers reported much higher concentrations in swine (up to 1.599 mg/kg) and ruminant feed (up to 0.907 mg/kg) together with Cavaglieri and colleagues in cow feed (range 1.200–3.060 mg/kg) [57,62]. These studies confirm that, occasionally, ZEN contamination may rise up to exceptionally high levels compared to the concentration usually encountered in feed samples. Average ZEN levels detailed in this work are quite congruent with data obtained in other European countries such as Poland [63], Norway [64], Slovakia [65] and Spain [16,66] whereas extra-EU findings seem to report higher contamination levels in terms of incidence and overall concentration [61,62,67,68].

### 4.3. Ochratoxin A

Incidence of OTA contaminated samples (0.7%) was extremely lower than AFB_1_ (5.3%) and ZEN (9.6%). This finding may be addressed to a high presence of left-censored data.

Pozzo and coworkers [69] analyzed 30 feed samples from different swine farms in North-western Italy detecting OTA from 0.0002 to 0.0384 mg/kg; Pietruszka and coworkers 2017 performed a similar field experiment sampling 300 swine feed samples from Polish farms from 2014 to 2016 [70]. The average OTA concentration in incurred samples was 0.0027 mg/kg with five out of nine incurred samples with OTA levels <0.010 mg/kg. On these bases, the majority of Pozzo and Pietruszka samples would have tested negative (<LOQ) if analyzed with our method thus confirming the left-censored data hypothesis, also considering that the limit of quantitation of OTA in feeds was set at 0.025 mg/kg. Moreover, Binder and colleagues [71] further demonstrated that OTA contamination in feed samples recovered in Southern Europe and the Mediterranean area (representative of Umbria and Marche Regions), is generally low (0.006 mg/kg). In our monitoring study, 26 cereal-based dog feeds were analyzed and in none of these samples was OTA found above LOQ (0.0040 mg/kg). Literature reports scarce OTA incidence in dog feed samples with concentration usually lower than the limit of quantitation of our method [72,73] with the exception of Gazzotti and coworkers [74] which analyzed 24 standard (cheaper) and 24 premium (more expensive) dog extruded feeds. Authors also spotted a significant difference in terms of OTA contamination between standard (0.0238 mg/kg) and premium feeds’ (0.0130 mg/kg) average contamination levels. 

### 4.4. General Considerations and Future Perspectives

The results of our monitoring study may help agricultural stakeholders and food safety personnel open up a few points of reflection for the upcoming years.

Mycotoxins contamination seems to be much more common in feed rather than in feed materials. Incidence rates revolve around 30% with no great difference between the most represented animal species admitting, although, a few exceptions that deserve a short insight. Rabbit feed, for example, seems to be more prone to zearalenone contamination even though only a limited number of samples was analyzed in this study (*n* = 19). Rabbit meat consumption has a strong tradition in Mediterranean countries [75] and, more recently, rabbits have become, after dogs and cats, one of the most common pets in European households. Rabbits that were exposed to subchronical doses of ZEN showed increased levels of hepatocellular and renal damage markers and changes in caecal microbiota [76,77]. This could pose some health risks especially in rabbit pets, which can live up to 10 years that may experience the effects of chronic exposure to ZEN through consumption of contaminated feed. Based on the actual knowledge, European Food Safety Authority (EFSA) could not exclude concerns on rabbit susceptibility to ZEN exposure [78]. Meat consumption is expected to grow in the upcoming years, mainly driven by income increase and population growth all over the world, with a robust shift towards poultry (+17.8%). In lower income countries, this reflects the better affordability of poultry compared to other meats while in richer countries this indicates an augmented preference for white meats, which are perceived to be easier to prepare as well as a healthier protein source [79]. Consequently, the demand of poultry feed production may rise as well, and a more thorough control program may be the right choice to assure feed quality whilst preventing economic losses deriving from animal deaths and reduced egg production [14,15,22,80]. Maize is often contaminated by AFB_1_ so is not surprising to find this mycotoxin in all types of feed, that are often formulated using maize as primary carbohydrates source. Cattle feed, although, is often produced using cottonseed (up to 15%) as a protein source [81]. Cottonseed is reported to be often contaminated by aflatoxins with levels up to 200 ng/g and its inclusion in cattle feeds may partially explain why this type of feed has the highest average levels of AFB_1_ [82,83]. Co-occurrence of mycotoxins in samples was rarely reported in our study (0.8% of the analyzed samples). Possible synergistic effects are documented by literature [84] although in vitro models often result in conflicting data [85]. Combined toxicity effects are influenced by a series of experimental parameters (cell types, exposure time and statistical analysis models) including that many studies are performed at high concentrations lacking information about sub-toxic mycotoxin levels exposure, which is more coherent with consumption of contaminated feed and food. On these bases, it is not possible to formulate any hypotheses on the relevance of multimycotoxin contamination for feed and feed materials and its impact on animal and human health. In the upcoming years climate change will very likely exacerbate extreme and unusual weather conditions that will directly enhance the possibility that mycotoxin contamination may rise as well. The possibility to integrate weather data with mycotoxin contamination of crops in order to build a predictive model for mycotoxin occurrence in feed started in the early 2000s with DONcast^®^ [86], for the prediction of deoxinivalenol levels in Canadian wheat. More recently, evaluations based on mechanistic models [87,88] and machine learning [89] have been carried out. Predictive tools are built using the high occurrence data obtained by multiannual monitoring of mycotoxin in crops and can be useful to understand mycotoxins contamination patterns based on climate and to deploy focused feed analysis programs. Liu and coworkers reported interesting results developing predicting models for aflatoxin (PREMA) and fumonisins (PREFUM) in maize in a case study conducted from 2012 to 2018 in Serbia. The developed models showed a prediction contamination accuracy around 80% for both classes of mycotoxins based on geographical localization and weather conditions [88]. These tools, if furtherly improved, may be very useful in the future for food and feed stakeholders as well as food safety authorities to take rapid decisions to contain mycotoxin contamination of crops.

## 5. Conclusions

Generally low incidence and concentrations of mycotoxins in feed and feed materials are attributable to the good effort made by farmers and growers to recognize and apply good agricultural practice (GAP) and good manufacturing practices (GMP) proposed by international monitoring organizations [90,91]. The data obtained from this study, although, highlights the importance of monitoring programs for animal feed in order to ensure quality of animal byproducts, animal well-being and to avoid commercial losses derived by potential mycotoxicoses outbreaks. Co-occurrence of at least two mycotoxins was observed in 10 out of 1443 of the analyzed samples (3 cattle feed, 2 cow feed, 2 sheep feed, 2 swine feed and 1 other species feed). A simultaneous presence of AFB_1_, ZEN and OTA was never observed. Drawing a conclusion on the hazards of multimycotoxin contamination and the effects on animal health is not possible at the present time as more in-depth in vivo studies are required. Non-compliant samples incidence was, on average, around 2% for feeds and feed materials. This parameter rises almost three-fold for maize samples (5.8%) suggesting how this matrix is probably far more sensitive to fungal development and proliferation compared to other feed materials. Nevertheless, the low non-compliant incidence highlighted by this study suggests that feed and feed materials can be considered, on average, safe. This assumption may change in the near future: climate change may worsen mycotoxin contamination of crops and the use of powerful predictive tools for mycotoxin occurrence together with a more thorough on-field education for farmers, producers and all the population may help food safety expert restrain and control this upcoming issue.

## Figures and Tables

**Table 1:** Relevant maximum contents and guidance levels for AFB_1_, ZEN and OTA in animal feed and feed materials.

	Products Intended for Animal Feed		Maximum Content ^1^ (mg/kg)
AFB_1_	Feed materials		0.02
	Complementary and complete feed		0.01
	Compound feed for dairy cattle and calves, dairy sheep and lambs, dairy goats and kids, piglets and young poultry animals		0.005
	Compound feed for cattle (except dairy cattle and calves), sheep (except dairy sheep and lambs), goats (except dairy goats and kids), pigs (except piglets) and poultry (except young animals)		0.02
	Products intended for animal feed		Guidance Value ^2^ (mg/kg)
ZEN	Feed materials	Cereals and cereal products with the exception of maize by-products	2
		Maize by-products	3
	Compound feed for:	Piglets, gilts (young sows), puppies, kittens, dogs and cats for reproduction	0.1
		Adult dogs and cats other than for reproduction	0.2
		Sows and fattening pigs	0.25
		Calves, dairy cattle, sheep (including lamb) and goats (including kids)	0.5
OTA	Feed materials	Cereals and cereal products	0.25
	Compound feed for:	Pigs	0.05
		Poultry	0.1
		Cats and dogs	0.01

^1^: Directive 2002/32/EC; ^2^: Commission Recommendation (EU) No. 1319/2016.

**Table 2:** Accuracy and precision in repeatability (RSD_r_) and intralaboratory reproducibility (RSD_wR_) conditions for feed.

	LOQ (mg/kg)	Matrix	Level (mg/kg)	Recovery (%)	s_r_	RSD_r_
AFB_1_	0.0020	Compound feed (BCR 375)	0.0020	103	5.8	5.7
		Cow feed	0.0049	89	4.4	5.0
		Chicken feed	0.0101	100	3.0	3.0
		Horse feed	0.0115	91	11	12
		Swine feed	0.0230	97	3.9	4.1
		Matrix	Level (mg/kg)	Recovery (%)	s_WR_	RSD_wR_
		Compound feed (ERM-BE375)	0.0020	92	15	16
	LOQ (mg/kg)	Matrix	Level (mg/kg)	Recovery (%)	s_r_	RSD_r_
ZEN	0.050	Compound feed (BCR 375)	0.050	100	9.5	9.5
		Swine feed	0.106	106	7.2	6.8
		Chicken feed	0.247	98	2.6	2.6
		Horse feed	0.247	109	11	11
		Cow feed	0.500	89	5.2	5.9
		Matrix	Level (mg/kg)	Recovery (%)	s_WR_	RSD_wR_
		Compound feed (ERM-BE375)	0.050	84	14	16
	LOQ (mg/kg)	Matrix	Level (mg/kg)	Recovery (%)	s_r_	RSD_r_
OTA	0.0040	Dog feed	0.0040	90	6.4	7.1
	0.025	Compound feed (BCR 375)	0.025	72	3.5	4.9
		Swine feed	0.053	92	2.9	3.1
		Chicken feed	0.101	76	2.1	2.8
		Horse feed	0.105	85	10	12
		Cow feed	0.103	84	2.4	3.9
	LOQ (mg/kg)	Matrix	Level (mg/kg)	Recovery (%)	s_WR_	RSD_wR_
	0.0040	Dog feed	0.0040	81	9.4	11
	0.025	Compound feed (ERM-BE375)	0.025	79	10	13

**Table 3:** Accuracy and precision in repeatability (RSD_r_) and intralaboratory reproducibility (RSD_wR_) conditions for feed materials.

	LOQ (mg/kg)	Matrix	Level (mg/kg)	Recovery (%)	s_r_	RSD_r_
AFB_1_	0.0080	Rapeseed	0.0084 (LOQ)	73	4.6	6.5
		Maize	0.0201	66	3.8	5.7
		Maize	0.0401	70	4.0	5.7
		Matrix	Level (mg/kg)	Recovery (%)	s_WR_	RSD_wR_
		Maize	0.0080 (LOQ)	72	14	22
		Barley	0.0080 (LOQ)	84	14	16
	LOQ (mg/kg)	Matrix	Level (mg/kg)	Recovery (%)	s_r_	RSD_r_
ZEN	0.200	Rapeseed	0.200 (LOQ)	76	13	17
		Maize	3.030	89	1.7	1.9
		Maize	5.998	86	2.6	3.1
		Matrix	Level (mg/kg)	Recovery (%)	s_WR_	RSD_wR_
		Maize	0.267 (LOQ)	81	8.6	11
		Barley	0.200 (LOQ)	84	11	14
	LOQ (mg/kg)	Matrix	Level (mg/kg)	Recovery (%)	s_r_	RSD_r_
OTA	0.100	Rapeseed	0.102 (LOQ)	83	3.7	4.5
		Maize	0.247	89	2.0	2.2
		Maize	0.509	90	2.4	2.7
		Matrix	Level (mg/kg)	Recovery (%)	s_WR_	RSD_wR_
		Maize	0.100 (LOQ)	75	5.7	7.5
		Barley	0.100 (LOQ)	86	7.3	7.5

**Table 4:** Feed and feed materials sampling and incidence rates.

Animal Species/ Feed Material	Samples	Detected Samples ^1^ (% of Samples)	Above Maximum Content/ Guidance Value (% of Samples)
Feed			
Cattle	194	48 (25%)	4 (2.1%)
Cow	54	15 (28%)	-
Dog	26	-	-
Poultry	62	10 (16%)	1 (1.6%)
Rabbit	19	10 (53%)	-
Sheep	261	58 (22%)	10 (3.1%)
Swine	179	62 (35%)	1 (0.6%)
Other species	31	9 (29%)	-
TOT	826	211 (26%)	16 (1.9%)
Feed materials			
Barley	186	-	-
Maize	240	35 (15%)	14 (5.8%)
Oat	44	-	-
Triticale	30	-	-
Wheat	61	-	-
Other materials	56	1 (0.2%)	-
TOT	617	36 (5.8%)	14 (2.2%)

^1^: at least one mycotoxin above LOQ.

**Table 5:** Mycotoxin levels in feed and feed materials.

Analyte	Species/Materials	Incidence (%)	Concentration (mg/kg)		
			Minimum	Maximum	Average
AFB_1_	Cattle	14	0.0025	0.1045	0.0133
	Cow	11	0.0036	0.0179	0.0072
	Poultry	8	0.0030	0.0291	0.0107
	Rabbit	5	0.0022		
	Sheep	5	0.0021	0.0216	0.0061
	Swine	13	0.0021	0.0138	0.0045
	Other species	10	0.0024	0.0040	0.0031
	Maize	15	0.0085	0.1234	0.0349
	Other feed materials	0.2	0.0156		
ZEN	Cattle	11	0.059	6.420	0.527
	Cow	20	0.057	1.017	0.204
	Poultry	8	0.058	0.330	0.196
	Rabbit	47	0.058	5.723	0.765
	Sheep	17	0.056	5.387	0.602
	Swine	25	0.051	1.698	0.182
	Other species	16	0.054	0.189	0.114
	Maize	0.2	0.668		
OTA	Cattle	1	0.034	0.042	0.039
	Cow	2	0.036		
	Sheep	1	0.038	0.085	0.061

## Data Availability

The data presented in this study are available on request from the corresponding author.

## Supplementary Materials

The following supporting information can be downloaded at: https://www.mdpi.com/article/10.3390/foods13020313/s1, Figure S1. Overall incidence of feed and feed materials from 2018 to 2022; **Table S1**. LC gradient and FLD parameters; Table S2. Linearity of Aflatoxin B_1_, Zearalenone and Ochratoxin A determined with back-calculated concentration (BCC); Table S3. LODs and LOQs for Aflatoxin B_1_, Zearalenone and Ochratoxin A; Table S4. Proficiency tests.
