# Supplementary material for: Co-Occurrence of Aflatoxin B1, Zearalenone and Ochratoxin A in Feed and Feed Materials in Central Italy from 2018 to 2022
Source: Foods. 2024 Jan 18;13(2):313. doi: 10.3390/foods13020313 (PMC10815256; doi:10.3390/foods13020313)
Supplement: Supplementary file 1 [file foods-13-00313-s001.zip › foods-2801419-supplementary.pdf]

Supplementary material

Figure S1. Overall incidence of feed and feed materials from 2018 to 2022.

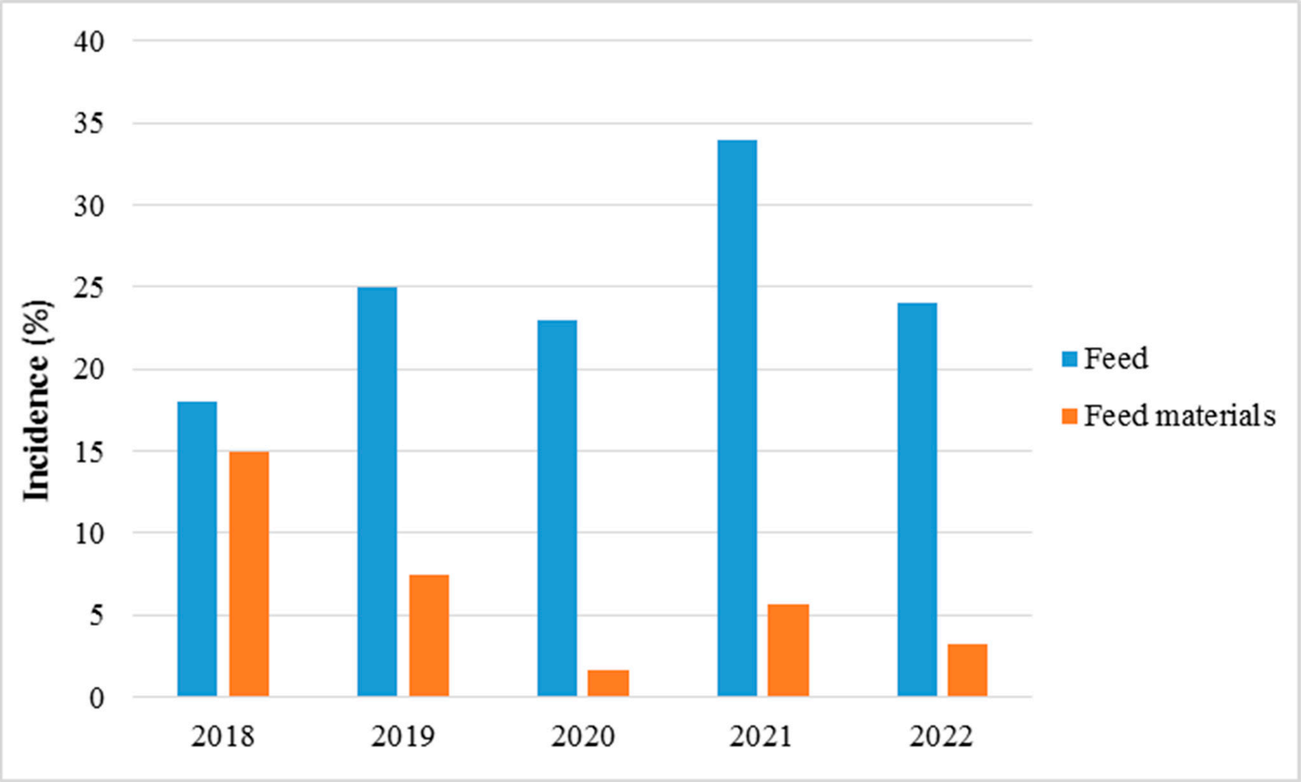

Table S1. LC gradient and FLD parameters

<table>
<tr><th>Time</th><th>MeOH (%)</th><th>ACN (%)</th><th>AcOH (%)</th><th>Flow (mL/min)</th></tr>
<tr><td>0.00</td><td>30</td><td>0</td><td>70</td><td rowspan="4">0.6</td></tr>
<tr><td>3.50</td><td>30</td><td>0</td><td>70</td></tr>
<tr><td>3.60</td><td>1</td><td>29</td><td>70</td></tr>
<tr><td>5.00</td><td>1</td><td>29</td><td>70</td></tr>
<tr><td>6.00</td><td>1</td><td>29</td><td>70</td><td rowspan="4">1.0</td></tr>
<tr><td>9.60</td><td>1</td><td>29</td><td>70</td></tr>
<tr><td>11.00</td><td>1</td><td>29</td><td>70</td></tr>
<tr><td>11.10</td><td>30</td><td>0</td><td>70</td></tr>
<tr><td>13.60</td><td>30</td><td>0</td><td>70</td><td rowspan="2">0.6</td></tr>
<tr><td>15.50</td><td>30</td><td>0</td><td>70</td></tr>
<tr><td colspan="5">Fluorescence detector parameters</td></tr>
<tr><td></td><td>λ_{ecc} (nm)</td><td>λ_{em} (nm)</td><td>Gain</td><td>Sensitivity</td></tr>
<tr><td>AFB_1</td><td>364</td><td>434</td><td>x16</td><td>High</td></tr>
<tr><td>ZEN</td><td>270</td><td>470</td><td>x4</td><td>High</td></tr>
<tr><td>OTA</td><td>334</td><td>460</td><td>x16</td><td>Medium</td></tr>
</table>

Table S2. Linearity of Aflatoxin B_1, Zearalenone and Ochratoxin A determined with back-calculated concentration (BCC)

**Aflatoxin B1**			
**µg/mL**	**Area**	**BCC (µg/mL)**	**Δ%**
0	0		
0.001	967770	0.0010	**0.8**
0.0026	2385680	0.0026	**-1.5**
0.0051	4742022	0.0051	**0.7**
0.0102	9458215	0.0103	**1.0**
0.0204	18640660	0.0203	**-0.3**
**Slope**	913911860		
**Intercept**	46268		

**Zearalenone**			
**µg/mL**	**Area**	**BCC (µg/mL)**	**Δ%**
0	0		
0.0284	805717	0.0278	**-2.2**
0.1136	3164323	0.1152	**1.4**
0.2841	7752185	0.2853	**0.4**
0.5682	15397862	0.5688	**0.1**
1.1363	30681720	1.1355	**-0.1**
**Slope**	26969894		
**Intercept**	56625		

**Ochratoxin A**			
**µg/mL**	**Area**	**BCC (µg/mL)**	**Δ%**
0.0000	0		
0.0129	777581	0.0127	**-1.9**
0.0322	1920707	0.0323	**0.2**
0.0643	3810293	0.0647	**0.6**
0.1286	7606621	0.1298	**1.0**
0.2573	14983463	0.2564	**-0.3**
0.5145	30027207	0.5146	**0.0**
**Slope**	58275874		
**Intercept**	39842		

Table S3. LODs and LOQs for Aflatoxin B_1, Zearalenone and Ochratoxin A

Analyte	Theoretical LOD (mg/Kg)	Theoretical LOQ (mg/Kg)	Practical LOQ in feed (mg/Kg)	Practical LOQ in feed materials (mg/Kg)
Aflatoxin B_1	0.0005	0.0015	0.0020	0.0080
Zearalenone	0.017	0.051	0.050	0.200
Ochratoxin A	0.010	0.031	0.025	0.100
Ochratoxin A (dog feed)	0.0014	0.0043	0.0040	-

Table S4. Proficiency tests

Proficiency test	Matrix	Analyte	Assigned value (mg/kg)	Submitted value (mg/kg)	z-score
Progetto Trieste – F1962 (October 2019)	Feed	AFB_1	0.01207	0.01605	1.18
FAPAS 04430 (December 2021)	Feed	AFB_1	0.0191	0.0223	0.8
		ZEN	0.0126	0.0110	-0.6
		OTA	0.0745	0.0820	0.5
FAPAS 04447 (July 2022)	Feed	AFB_1	0.0109	0.0068	-1.7
		ZEN	0.116	0.081	-1.4
		OTA	0.0100	0.0102	0.1
